# Leaf electrophysiological readouts reveal bicarbonate-associated shifts in early water-deficit response modes in *Broussonetia papyrifera* and *Morus alba*

**DOI:** 10.1080/15592324.2026.2657093

**Published:** 2026-04-09

**Authors:** Lei Fang, Yanyou Wu, Cheng Zhang, Shihao Zhang

**Affiliations:** a School of Public Health, Guiyang Kangyang University, Guiyang, China; b State Key Laboratory of Environmental Geochemistry, Institute of Geochemistry, Chinese Academy of Sciences, Guiyang, China; c School of Public Health, Guizhou Medical University, Guizhou, China

**Keywords:** Karst, leaf physiology, electrical properties, combined effects, seedling establishment

## Abstract

Karst carbonate regions are characterized by shallow soils and high bicarbonate availability, and seedlings during establishment may experience episodic water deficit. However, it remains unclear whether bicarbonate co-occurrence alters early response modes to short-term water deficit in a species-dependent manner. Here, we compared *Broussonetia papyrifera* and *Morus alba* under three treatments for 10 d: control (CK), polyethylene glycol–induced water deficit (WD), and WD with bicarbonate addition (WD + Bic). By integrating photosynthetic traits with non-destructive leaf electrophysiological signals, we evaluated early responses at both leaf and cellular scales. Under WD + Bic, the net photosynthetic rate, electrophysiologically derived nutrient-use-efficiency, and metabolic-energy indices of *B. papyrifera* were closer to the CK than those under WD. In contrast, *M. alba* deviated less from CK under WD alone, yet net photosynthetic rate and energy-related indices remained below CK under WD + Bic, accompanied by a stepwise decline in intrinsic water conductivity (CK > WD > WD + Bic). Overall, WD + Bic elicited species-specific shifts in early response trajectories relative to WD alone, rather than a uniform intensification of stress responses. These electrophysiological readouts helped distinguish distinct response modes, supporting electrophysiological phenotyping as a non-destructive candidate approach for early screening and species–habitat matching under carbonate-rich conditions.

## Introduction

Karst ecosystems are highly sensitive to environmental fluctuations because of their shallow soils and the intense weathering of carbonate bedrock.^1^ In these regions, vegetation commonly experiences recurrent droughts caused by rapid water loss, together with high bicarbonate levels derived from carbonate weathering.^2^ Under such settings, bicarbonate stress may co-occur with episodic drought or rapid substrate water loss, creating a combined stress scenario during early growth stages.^3^ Co-occurring environmental factors are not necessarily additive; instead, their interactions can generate non-additive physiological effects and reshape plant response trajectories.^4^^,^^5^ Plants typically respond to water deficit through stomatal regulation, the accumulation of osmotic adjustment compounds, and the activation of antioxidant systems.^6-8^ In contrast, bicarbonate primarily disrupts ion homeostasis and impairs membrane function.^9^^,^^10^ Previous studies have suggested that bicarbonate availability may influence carbon acquisition or photosynthetic adjustment under certain conditions in some karst species.^11^ Therefore, in the context of projected drought intensification,^12^ it is essential to adopt a compound-stress perspective in karst environments and to examine early response patterns of woody seedlings under short-term water deficit with and without bicarbonate co-occurrence, rather than inferring adaptation solely from single-stress scenarios.

Because the seedling establishment stage is highly sensitive to environmental fluctuations,^13^ early identification of response differences among woody species under the co-occurrence of water deficit and bicarbonate is important for understanding species adaptive strategies and species–habitat matching. *Broussonetia papyrifera* (*Bp*) is highly adaptable and widely used in the vegetation restoration of degraded land.^14^ It also has potential applications in forage production, fiber utilization, and medicinal uses.^15^^,^^16^ In contrast, *Morus alba* (*Ma*) is an important economic and fodder tree species with additional potential in soil remediation.^17^^,^^18^ Previous studies have reported that *Ma* can maintain relatively stable growth under water deficit conditions.^19^ Although both *Bp* and *Ma* have significant ecological and economic value, their research focuses differ. Therefore, we selected *Bp* and *Ma* as contrasting species and focused on bicarbonate co-occurrence under short-term water deficit to test whether this compound constraint reshapes early response modes in a species-specific manner.

To date, most studies have relied mainly on conventional physiological indicators, such as stomatal conductance, photosynthetic rate, and water potential, which have been widely used to evaluate the response patterns of plants.^20^^,^^21^ However, combined stresses often exert their effects first at the cellular level,^22^ where early responses are difficult to capture using conventional physiological indicators. Conventional methods can provide integrative information on whole-plant responses but are generally time-consuming and some assays require destructive sampling. In contrast, electrophysiological techniques can capture dynamic changes at the cellular level.^23^ When the environment changes, the response of plants to these changes manifests more slowly in the overall plant structure, but their perception of the environment in terms of electrical potential activity is very rapid.^24^ In signaling terms, leaf electrical properties can be viewed as integrative readouts of membrane polarization and ion-transport activity, providing a rapid window into how plants encode and coordinate responses to compound cues.^25^ Therefore, approaches capable of detecting real-time, cellular-level responses are particularly valuable for resolving early stress dynamics.

Our previously developed parallel-plate capacitance system enables noninvasive detection of leaf electrical properties and their stress responses.^26^ Based on Gibbs free energy and the Nernst equation, a quantitative model can be established to describe the relationships between capacitance, resistance, impedance, and reactance and the clamping force applied by the sensor.^27^ The plasma membrane of leaf mesophyll cells exhibits strict selective permeability to ions, ion groups, and electric dipoles. As a result, the electrolyte solutions on both sides of the membrane form a characteristic conductive state, which is analogous to the two electrodes of a capacitor, while the membrane itself functions as the dielectric layer. Intracellular compartments such as the vacuole and cytoplasm contribute resistive components, and mesophyll cells can therefore be approximated as a concentric spherical capacitor with inductive and resistive elements. By applying external pressure to the leaf and thereby altering the membrane state of mesophyll cells, electrical signals can be recorded under different stimuli. These signals may be associated with differences in plant response states under changing environmental conditions, as well as the balance of intracellular water status.^28^ These environmental changes inherently alter the tissue's electrolyte concentrations and water content, which modifies the dielectric polarization and ionic conductivity of the leaf. Such cellular-level changes may contribute to variation in the measured electrical parameters. Based on these physical measurements, specific leaf electrophysiological indicators were further derived to evaluate the plants.

In carbonate-dominated karst landscapes, carbonate buffering often leads to elevated bicarbonate concentrations in soil and pore water, which may co-occur with episodic water deficit during seedling establishment. However, it remains unclear how bicarbonate co-occurrence modifies early responses to water deficit and whether such modulation differs between woody species. Here, we compared *Bp* and *Ma* under three treatments: control, polyethylene glycol–induced water deficit, and water deficit with bicarbonate addition. We integrated conventional physiological measurements with electrophysiological readouts to address three questions: (i) does bicarbonate co-occurrence shift the trajectory and extent of early responses relative to water deficit alone? (ii) do the two species exhibit distinct adjustment patterns under combined stress? and (iii) can non-destructive electrophysiological indicators help detect and distinguish these differences at an early stage by capturing cellular-scale response states under bicarbonate co-occurrence?

## Materials and methods

### Plant materials

Seeds of *Bp* were collected from mature trees in Guizhou Province, China, while seeds of *Ma* were obtained from a commercial seed supplier in Jiangsu Province, China. Seeds of both species were surface-sterilized with 75% ethanol for 1 min, rinsed thoroughly with distilled water, and soaked in distilled water for 24 h prior to germination. After the emergence of true leaves, seedlings were transplanted into plastic pots (8.5 cm in diameter, 9.7 cm in height) filled with a vermiculite–perlite substrate (1:2, v/v). Plants were grown under controlled environmental conditions with a 12 h light/12 h dark photoperiod, a photosynthetically active radiation of 500 ± 50 μmol m^−2^ s^−1^, d/night temperatures of 25 °C ± 2 °C/20 °C ± 2 °C, and a relative humidity of 50%–55%. Each pot was placed in a seedling tray, allowing nutrient solution to be supplied from the bottom of the container. Prior to stress treatments, seedlings were grown under 1/2-strength modified Hoagland nutrient solution (Table S1), which was renewed regularly. Seedlings of *Bp* and *Ma* were cultivated until the plants reached approximately 22 cm in height, after which seedlings with consistent growth were selected for the experiments.

### Experimental design

To simulate bicarbonate-rich conditions that can coincide with episodic water deficit during early seedling establishment, we compared a water-deficit treatment with and without added NaHCO_3_ against a control. There were three treatment groups in the experiment. The plants in the control group (CK) were cultivated with 1/2-strength modified Hoagland nutrient solution for 10 d. In the water deficit group (WD), plants were cultivated for 10 d in 1/2-strength modified Hoagland nutrient solution supplemented with polyethylene glycol 6000 (PEG6000; 80 g L^−1^) to induce water deficit. PEG6000 was used as an osmotic agent to impose a short-term reduction in water availability during the seedling stage. The combined water deficit and bicarbonate group (WD + Bic) was subjected to simulated water deficit and bicarbonate stress in which the plants were cultivated with 1/2-strength modified Hoagland nutrient solution supplemented with 80 g L^−1^ PEG6000 and 10 mM NaHCO_3_ for 10 d. This three-treatment design was not intended to isolate bicarbonate main effects; instead, we focused on bicarbonate-associated differences by directly contrasting WD and WD + Bic. A 10-d exposure was selected to capture early-stage responses.^29^^,^^30^ Each treatment consisted of three biological replicates (n = 3) per species, with each replicate representing an individual plant.

The concentration of PEG6000 (80 g L^−1^), widely used to simulate a moderate-to-strong osmotic constraint in hydroponic and controlled-environment studies, results in an osmotic potential of approximately −0.10 MPa.^31^^,^^32^ NaHCO₃ was applied at 10 mM to impose a bicarbonate challenge under controlled conditions, corresponding to approximately 10 meq L^−1^ HCO₃^−^. The solution pH of the three treatments was adjusted to 7.7 ± 0.1. Treatment solutions were renewed daily throughout the experimental period, thereby maintaining relatively stable pH conditions during exposure. Each pot received 250 mL of fresh treatment solution per day.

### Leaf gas exchange, water potential, and growth measurements

The photosynthetic indices of the plants were measured between 8:00 and 10:00 a.m. on Days 0, 2, 4, 6, 8, and 10. The gas exchange parameters of the leaves, namely, the net photosynthetic rate (Pn), stomatal conductance (gs), and transpiration rate (Tr), were measured using a portable photosynthesis measurement system (Li-6400; LI-COR, Lincoln, NE). The PPFD was set at 500 μmol m^−2^ s^−1^ to match the light source, and the CO_2_ concentration was 380 μmol mol^−1^. Additionally, the leaf temperature was maintained at approximately 25 °C, the relative humidity within the chamber was kept at approximately 50%, and the air flow rate was set to 500 μmol s^−1^. A standard 2 × 3 cm leaf chamber was used, and measurements were conducted on the second fully expanded leaves. Water use efficiency (WUE) was calculated as follows:

(1) $$\mathrm{WUE}=\frac{\mathrm{Pn}}{\mathrm{Tr}}.$$

At the end of the experiment, the water potential (Ψ_*w*_) of the fourth fully expanded leaf was measured using a Psypro dewpoint psychrometer with a C-52 chamber. On Day 0, the second fully expanded leaf was tagged, and the same leaf length and plant height were measured with a ruler on Day 0 and Day 10 to calculate the daily growth rate of the plant.

### Measurement of leaf electrical parameters

Using a parallel-plate capacitance sensor (Figure S1), graded clamping forces were applied as external stimuli that transiently affect membrane permeability and solute distribution, thereby inducing measurable changes in leaf electrical parameters from which derived electrophysiological indices were subsequently calculated. Electrophysiological measurements were employed to capture cellular-level response characteristics. Electrical indicators of plant leaves were tested on Day 10 using an LCR-6100 tester (Gwinstek, Taiwan, China) to capture the integrated endpoint state of the early-acclimation stage and to align electrophysiological profiling with the endpoint measurements of water potential, growth, and leaf gas exchange. Fully expanded leaves were clamped between two parallel electrodes, and impedance parameters were recorded at a fixed frequency of 3 kHz and a test voltage of 1.5 V in parallel mode.

The LCR meter provided resistance (*R*), impedance (*Z*), and capacitance (*C*) values under a series of clamping forces (1 N, 2 N, 3 N, 4 N, and 5 N). For each plant, *R*, *Z*, and *C* were recorded across five clamping forces as technical measurements, and capacitive reactance (Xc) and inductive reactance (XL) were calculated at each force; all parameters were summarized per plant and analyzed at the biological-replicate level (n = 3). The Xc and XL were calculated as follows:

(2) $$\mathrm{Xc}=\frac{1}{2\pi \mathrm{fC}},$$

(3) $$\frac{1}{-\mathrm{XL}}=\frac{1}{\text{Z}}-\frac{1}{\text{R}}-\frac{1}{\text{Xc}}.$$

In Equation (2), *π* = 3.1416 and *f* = 3000 Hz. In accordance with previous work,^23^ the fitting equations describing the relationships between the clamping force and leaf electrical parameters were determined as shown in Equations (4)–(8):

(4) $$R=y_{0}+k_{1}e^{-b_{1}F},$$

(5) $$Z=p_{0}+k_{2}e^{-b_{2}F},$$

(6) $$\mathrm{Xc}=q_{0}+k_{3}e^{-b_{3}F},$$

(7) $$\mathrm{XL}=a_{0}+k_{4}e^{-b_{4}F},$$

(8) $$C=x_{0}+\mathrm{hF}.$$

The *y*_0_, *k*_1_, *b*_1_, *p*_0_, *k*_2_, *b*_2,_ *q*_0_, *k*_3_, *b*_3_, *a*_0_, *k*_4_, *b*_4_, *x*_0_, and *h* are fitted coefficients estimated from Equations (4)–(8); their values are listed in Supplementary Table S2. When the clamping force was absent (F = 0 N), the inherent conduction resistance (IR), intrinsic impedance (IZ), intrinsic capacitive reactance (IXc), and intrinsic inductive reactance (IXL) of the leaves were determined as described in Equations (9)–(12):

(9) $$\mathrm{IR}=y_{0}+k_{1},$$

(10) $$\mathrm{IXc}=q_{0}+k_{3},$$

(11) $$\mathrm{IXL}=a_{0}+k_{4},$$

(12) $$\frac{1}{\mathrm{IZ}}=\frac{1}{\mathrm{IR}}+\frac{1}{\mathrm{IXc}}-\frac{1}{\mathrm{IXL}}.$$

The intrinsic capacitance (IC) was calculated according to Equation (13):

(13) $$\mathrm{IC}=\frac{1}{2\pi \mathrm{fI}\mathrm{Xc}}.$$

In this study, “electrophysiological indices” refer specifically to descriptor variables derived from leaf electrical parameters measured in living tissues (*R*, *Z*, *C*, Xc, and XL) in combination with the clamping-force response model. These indices are used to characterize physiological-level variation in leaf electrical behavior, but should be interpreted as model-based physiological proxies rather than direct measurements of intracellular physiological state.

### Plant intracellular water

In hydrated mesophyll tissue, within this framework, the water-rich compartments (mainly vacuole and cytosol) largely determine the measurable C (reflecting water storage and retention), whereas changes in *R* and *Z* track hydration-dependent conductivity. Therefore, intracellular water traits were derived from electrophysiological measurements, and related frameworks have previously been applied in studies of *Coix lacryma-jobi L*.^33^ The intrinsic water conductivity (IWC), intracellular water-holding capacity (IWHC), intracellular water-holding time (IWHT), and dynamic water transfer rate (WTR) were calculated as follows:

(14) $$\mathrm{IWC}=\frac{0.5\mathrm{hf}}{1000},$$

(15) $$\mathrm{IWHC}=\sqrt{{\left(\mathrm{IC}\right)}^{3}},$$

(16) IWHT=IC×IZ,

(17) $$\mathrm{WTR}=\frac{\mathrm{IWHC}}{\mathrm{IWHT}}.$$

### Plant intracellular nutrient dynamics

Nutrient uptake and transmembrane transport are associated with membrane composition and transport proteins (channels and carriers), and related ion-flux and polarization states may be indirectly reflected in leaf electrophysiological properties. Accordingly, force-dependent variations in electrophysiological parameters were used to quantify intracellular nutrient-related traits, and the underlying framework has previously been tested across multiple woody and herbaceous species, including *B. papyrifera*, *Rhus chinensis*, *Toona sinensis*, *Ipomoea batatas*, *Senecio scandens*, *Solanum tuberosum*, and *Capsicum annuum*.^34^ Passive nutrient transport capacity (NPT), active nutrient transport capacity (NAT), nutrient use efficiency (NUE), nutrient transfer rate (NTR), active transport flow of nutrient (UAF), nutrient active translocation capacity (NAC), and the unit for nutrient-relative transport flow (UNF) were calculated as:

(18) $$\mathrm{NPT}=\frac{\frac{1}{\mathrm{IXc}}}{\frac{1}{\mathrm{IR}}},$$

(19) $$\mathrm{NAT}=\frac{\frac{1}{\mathrm{IXL}}}{\frac{1}{\mathrm{IR}}},$$

(20) $$\mathrm{NUE}=\frac{100}{\mathrm{NAT}+\mathrm{NPT}},$$

(21) $$\mathrm{NTR}=\frac{\sqrt{{\left(\mathrm{IC}\right)}^{3}}}{\mathrm{IC}\times \mathrm{IZ}},$$

(22) $$\mathrm{UAF}=\frac{\mathrm{IXc}}{\mathrm{IXL}},$$

(23) NAC=UAF×NTR,

(24) $$\mathrm{UNF}=\frac{\mathrm{IR}}{\mathrm{IXc}}+\frac{\mathrm{IR}}{\mathrm{IXL}}.$$

### Cellular metabolic energy

Metabolic energy was estimated from leaf electrophysiological parameters because cellular metabolism (largely ATP-supported) sustains ion transport and electrochemical gradients, which are reflected in measurable electrical properties of leaf tissue. Based on the *C*, *R*, and *Z* of plant leaves, the model constructed according to the electrophysiological parameters was used to calculate the metabolic energy of plant leaf cells, and related energy electrophysiological frameworks have previously been applied, for example, in *Brassica rapa*.^35^ Based on Equation (8), the specific effective thickness of the leaves (*d*) was calculated as:

(25) $$d=\frac{{\text{U}}^{2}h}{2}.$$

In Equation (25), U = 1.5 V. Based on Equations (4) and (5), unit-level energetic indices derived from *R* and *Z* were calculated as:

(26) $$\Delta G_{R-E}=\frac{\ln\;k_{1}-\;\ln\;y_{0}}{b_{1}},$$

(27) $$\Delta G_{Z-E}=\frac{\ln\;k_{2}-\;\ln\;p_{0}}{b_{2}}.$$

Metabolic energy of plant leaf cells based on *R* (Δ*G_R_*), metabolic energy of plant leaf cells based on *Z* (Δ*G_Z_*), and cellular metabolic energy of plant leaf (Δ*G*) were obtained:

(28) $$\Delta G_{R}=\Delta G_{R-E}d,$$

(29) $$\Delta G_{Z}=\Delta G_{Z-E}d,$$

(30) $$\Delta G=\frac{\Delta G_{R}+\Delta G_{Z}}{2}.$$

### Statistical analysis

SPSS software (v25; IBM Corp., Armonk, NY, USA) was used for statistical analyses. For time-course leaf gas-exchange traits, linear mixed-effects models (LMMs) were fitted separately for each species, with treatment, time, and their interaction as fixed effects, plant identity as a random intercept, and an AR(1) covariance structure to account for repeated measurements. For all endpoint indicators, treatment differences were examined on observed values using one-way ANOVA followed by Tukey's HSD within each species. Origin software (v9.4; OriginLab Corp., Northampton, MA, USA) was used for figure preparation. Differences were considered significant when *p* < 0.05. The data are expressed as the mean ± standard error (SE).

Principal component analysis (PCA) was performed in R software (v4.5; R Core Team). PCA was conducted using the PCA function in the *FactoMineR* package based on centered and scaled variables. Scores of the first two principal components were used for visualization. For each species × treatment combination, group centroids were calculated as the mean PC scores of replicate samples, and centroid shifts from the CK to WD and WD + Bic treatments were visualized as arrows in the PCA space.

## Results

### Leaf gas exchange and physiological indices

**Figure 1.**
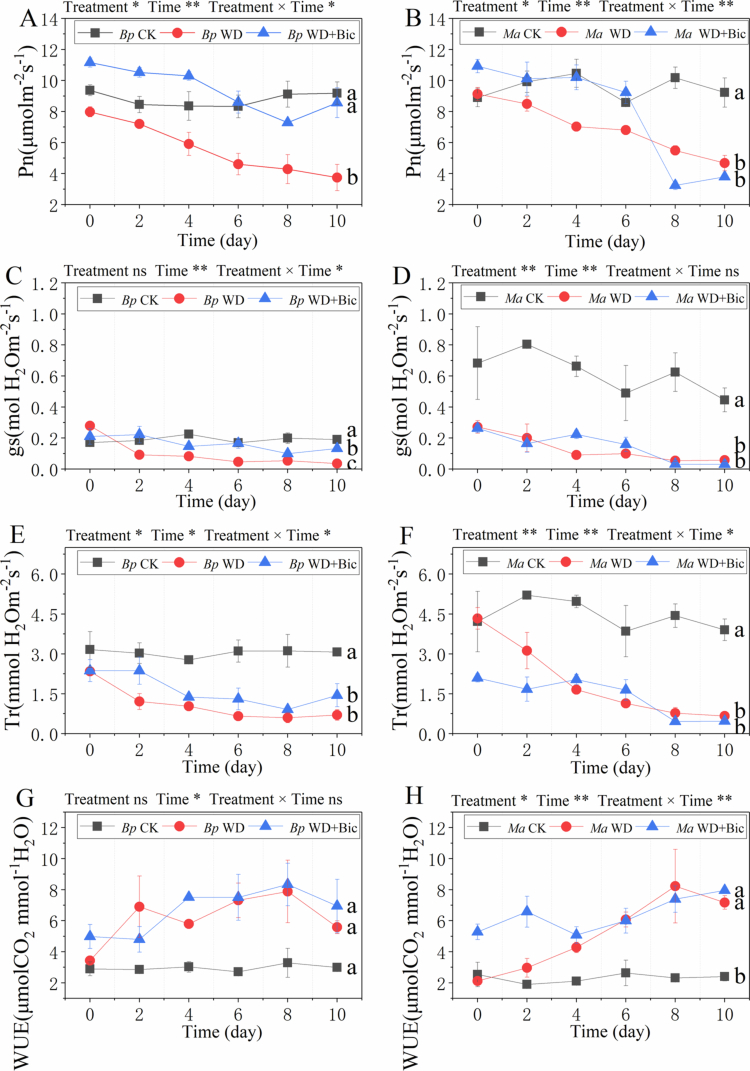
Temporal changes in leaf gas exchange parameters over a 10-d treatment period in *Broussonetia papyrifera* (*Bp*) and *Morus alba* (*Ma*) under three treatments: control (CK), water deficit (WD), and combined water deficit and bicarbonate (WD + Bic). Net photosynthetic rates of *Bp* (A) and *Ma* (B). Stomatal conductances of *Bp* (C) and *Ma* (D). Transpiration rates of *Bp* (E) and *Ma* (F). Water use efficiencies of *Bp* (G) and *Ma* (H). Asterisks shown above each panel indicate the significance of the overall fixed effects of treatment, time, and treatment × time based on Type III tests from linear mixed-effects models within each species (*p < 0.05, **p < 0.01; ns, not significant). Data are mean ± SE (n = 3). Different letters indicate significant differences among treatments at Day 10 within each species (one-way ANOVA followed by Tukey's HSD, *p* < 0.05).

Time-course photosynthetic traits showed treatment- and time-dependent trajectories over the 10-d period (Figure 1). In *Bp*, Pn exhibited significant effects of treatment, time, and their interaction. Under WD, Pn declined progressively from Day 0 to Day 10, whereas CK remained relatively stable. Under WD + Bic, Pn was maintained at a higher level than WD throughout the period; endpoint comparisons indicated that WD had a lower Pn than CK and WD + Bic (Figure 1A). Stomatal conductance (gs) was mainly time-dependent with a significant treatment × time interaction: gs decreased markedly under WD, and WD + Bic showed an intermediate trajectory between CK and WD; by Day 10, CK > WD + Bic > WD (Figure 1C). Tr showed a significant treatment × time interaction: Tr remained comparatively stable under CK but decreased under both WD and WD + Bic, with both stress treatments lower than CK at Day 10 (Figure 1E). WUE exhibited significant effects of time and increased under WD and WD + Bic relative to CK during the treatment period, and endpoint WUE was not separated among treatments (Figure 1G).

In *Ma*, Pn showed significant treatment, time, and treatment × time effects. CK maintained relatively high Pn throughout, WD and WD + Bic induced a gradual decline; both were lower than CK at the endpoint (Figure 1B). For gs, both treatment and time effects were detected: gs remained substantially higher in CK, whereas WD and WD + Bic stayed low; at Day 10, CK exceeded both WD and WD + Bic (Figure 1D). Tr showed significant treatment, time, and treatment × time effects: CK maintained higher Tr, while WD and WD + Bic declined to low levels by Day 10, with both stress treatments lower than CK (Figure 1F). WUE exhibited a pronounced treatment × time interaction: WD and WD + Bic displayed progressively higher WUE than CK, and by Day 10 both stress treatments exceeded CK (Figure 1H).

The *Bp* leaf water potential decreased progressively from CK to WD + Bic and further to WD (Table 1). Compared with that in CK, the leaf water potential in WD + Bic and WD decreased by 0.39 MPa and 0.77 MPa, respectively. With respect to *Ma* leaves, the water potential of WD and WD + Bic also decreased, with decreases of 0.73 MPa in WD and 1.01 MPa in WD + Bic compared with those in CK. The plant height and leaf growth rates of *Bp* decreased significantly in WD and WD + Bic (Table 1). In *Ma*, plant height growth rate under WD remained significantly higher than under WD + Bic. The leaf growth rate decreased significantly in WD and WD + Bic.

**Table 1.** Leaf water potential and growth parameters of *Broussonetia papyrifera* (*Bp*) and *Morus alba* (*Ma*) under three treatments: control (CK), water deficit (WD), and combined water deficit and bicarbonate (WD + Bic). Data are presented as mean ± SE (*n* = 3). Within each species, different superscript letters indicate significant differences among treatments (one-way ANOVA with Tukey's HSD, *p* < 0.05); values sharing the same letter are not significantly different.

		CK	WD	WD + Bic
*Bp*	Ψ_*w*_ (MPa)	−0.40 ± 0.01^a^	−1.17 ± 0.07^c^	−0.79 ± 0.05^b^
	Plant height growth rate (cm/d)	0.17 ± 0.01^a^	0.01 ± 0.00^b^	0.03 ± 0.00^b^
	Leaf growth rate (cm/d)	0.12 ± 0.02^a^	0.02 ± 0.00^b^	0.03 ± 0.02^b^
*Ma*	Ψ_*w*_ (MPa)	−0.47 ± 0.02^a^	−1.20 ± 0.09^b^	−1.48 ± 0.06^b^
	Plant height growth rate (cm/d)	0.44 ± 0.07^a^	0.26 ± 0.05^a^	0.04 ± 0.01^b^
	Leaf growth rate (cm/d)	0.11 ± 0.01^a^	0.03 ± 0.00^b^	0.03 ± 0.01^b^

### Intrinsic electrophysiological parameters

**Figure 2.**
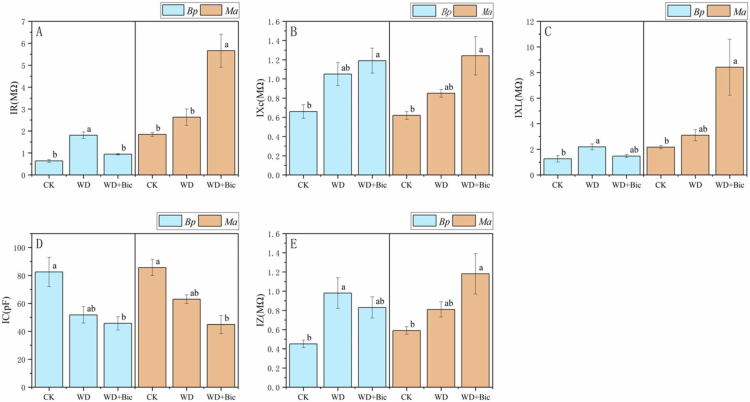
Intrinsic electrophysiological parameters of *Broussonetia papyrifera* (*Bp*) and *Morus alba* (*Ma*) under three treatments: control (CK), water deficit (WD), and combined water deficit and bicarbonate (WD + Bic). Data are mean ± SE (*n* = 3). Different letters indicate significant differences among treatments within each species (one-way ANOVA followed by Tukey's HSD, *p* < 0.05).

Figure 2 shows species-specific changes in intrinsic electrophysiological parameters across treatments. In *Bp*, WD significantly increased IR, IZ, and IXL relative to CK, whereas both tended to decline under WD + Bic, with IR returning to the CK level. IXc increased progressively and reached the highest value under WD + Bic, and IC was significantly reduced under WD + Bic compared with CK. In *Ma*, WD + Bic caused marked increases in IR, IXc, IXL, and IZ. Conversely, IC declined significantly under WD + Bic.

### Plant intracellular water

**Figure 3.**
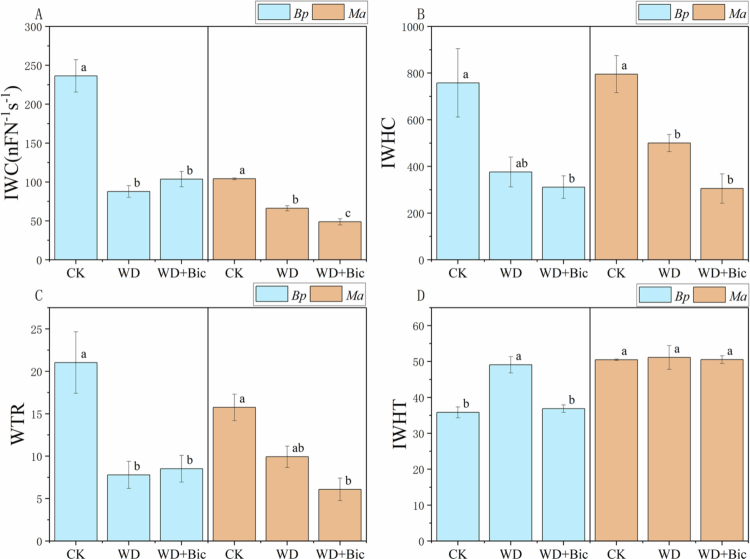
Plant intracellular water parameters of *Broussonetia papyrifera* (*Bp*) and *Morus alba* (*Ma*) under three treatments: control (CK), water deficit (WD), and combined water deficit and bicarbonate (WD + Bic). Data are mean ± SE (*n* = 3). Different letters indicate significant differences among treatments within each species (one-way ANOVA followed by Tukey's HSD, *p* < 0.05).

Figure 3 summarizes treatment effects on plant intracellular water indices. In *Bp*, IWC decreased significantly under WD and remained low under WD + Bic, while IWHC declined under WD + Bic compared with CK. WTR was markedly reduced by WD and stayed similarly low under WD + Bic. IWHT increased under WD but returned to the CK level under WD + Bic. In *Ma*, IWC showed a stepwise decrease from CK to WD and further to WD + Bic, and IWHC was significantly lower under both WD and WD + Bic than CK. WTR was significantly lower under WD + Bic than CK, whereas IWHT remained unchanged across treatments.

### Plant intracellular nutrient dynamics

**Figure 4.**
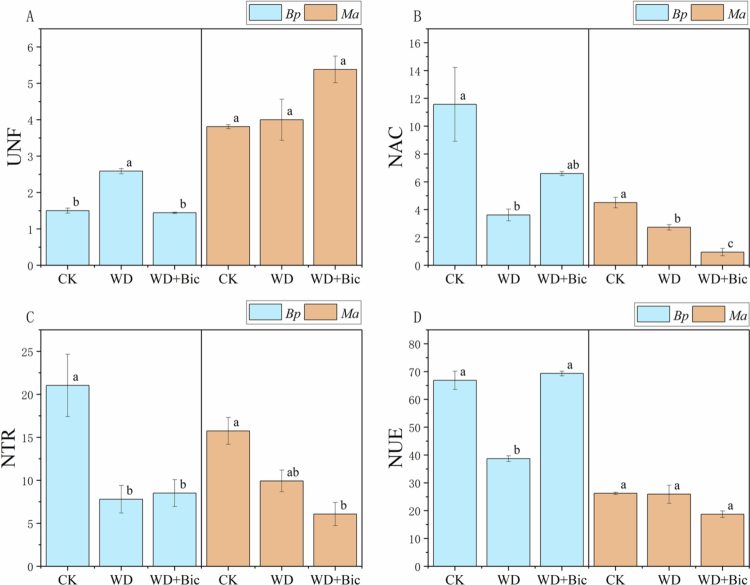
Nutrient transport parameters of *Broussonetia papyrifera* (*Bp*) and *Morus alba* (*Ma*) under three treatments: control (CK), water deficit (WD), and combined water deficit and bicarbonate (WD + Bic). Data are mean ± SE (*n* = 3). Different letters indicate significant differences among treatments within each species (one-way ANOVA followed by Tukey's HSD, *p* < 0.05).

Figure 4 shows treatment-dependent changes in plant intracellular nutrient dynamics. In *Bp*, UNF increased significantly under WD but returned to the CK level under WD + Bic. NAC decreased markedly under WD and partially recovered under WD + Bic, whereas NTR was significantly reduced under both WD and WD + Bic relative to CK. NUE declined under WD but recovered to the CK level under WD + Bic. In *Ma*, NAC decreased stepwise from CK to WD and further to WD + Bic. NTR declined significantly under WD + Bic. By contrast, UNF and NUE showed no significant differences among treatments.

### Cellular metabolic energy

**Figure 5.**
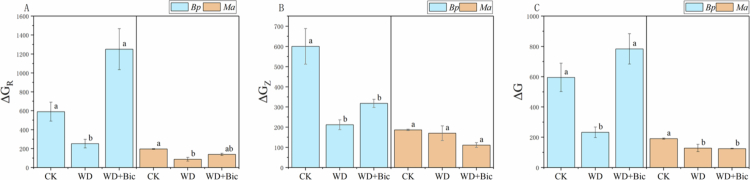
Cellular metabolic energy in *Broussonetia papyrifera* (*Bp*) and *Morus alba* (*Ma*) under three treatments: control (CK), water deficit (WD), and combined water deficit and bicarbonate (WD + Bic). Data are mean ± SE (*n* = 3). Different letters indicate significant differences among treatments within each species (one-way ANOVA followed by Tukey's HSD, *p* < 0.05).

Water deficit and bicarbonate markedly altered cellular metabolic energetic indices in both species, but with contrasting response patterns (Figure 5). In *Bp*, ΔG_R_ decreased under WD relative to CK and WD + Bic. For Δ*G_Z_*, CK was significantly higher than WD and WD + Bic. Δ*G* increased under WD + Bic relative to WD and was not significantly different from CK. In *Ma*, Δ*G_R_* also showed significant treatment effects: WD was significantly lower than CK, while WD + Bic was intermediate and not significantly different from CK or WD. For Δ*G_Z_*, no significant differences were detected among CK, WD, and WD + Bic. For Δ*G*, CK was significantly higher than both WD and WD + Bic, whereas WD and WD + Bic did not differ significantly from each other.

### Principal component analysis

**Figure 6.**
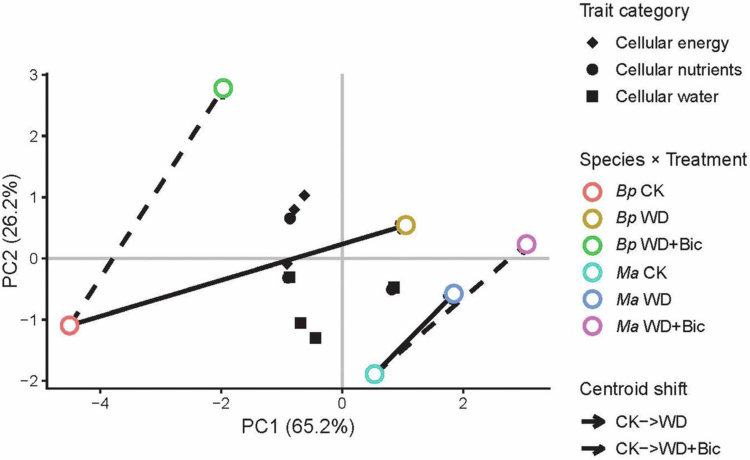
Principal component analysis (PCA) of electrophysiological traits in Broussonetia papyrifera (Bp) and Morus alba (Ma) under three treatments: control (CK), water deficit (WD), and combined water deficit and bicarbonate (WD + Bic). All the traits were further classified into three functional categories: (1) cellular energy (Δ*G_R_*, Δ*G_Z_*, and Δ*G*); (2) cellular nutrients (UNF, NUE, NTR, and NAC); and (3) cellular water (IWC, IWHC, IWHT, and WTR). Solid and dashed arrows denote centroid shifts relative to the corresponding CK centroid (CK → WD and CK→WD + Bic, respectively).

Figure 6 shows a PCA integrating cellular water, nutrient, and energy traits. PCA of electrophysiological traits explained 91.4% of the total variance on the first two axes (PC1 = 65.2%, PC2 = 26.2%). The score plot revealed clear separation between species × treatment groups, with *Bp* and *Ma* occupying distinct regions in the PC space. In *Bp*, the centroid shifted from CK toward WD along the positive PC1 direction, whereas the WD + Bic centroid moved away from WD and separated mainly along PC2. In *Ma*, centroids under WD and WD + Bic moved away from CK in a similar direction, with WD + Bic exhibiting a larger displacement than WD. The three functional trait categories largely overlapped around the central PCA region and did not form distinct module-specific clusters. Differences between the two species were captured mainly by the centroid shifts across treatments.

## Discussion

### Photosynthetic performance and early growth

Photosynthetic and growth-related traits revealed clear species-dependent divergence in early gas-exchange trajectories under water deficit when bicarbonate co-occurred. Under WD and WD + Bic stress, *Bp* and *Ma* exhibited distinct photosynthetic response patterns. In *Bp*, the temporal dynamic changes in Pn, Tr, and gs differed among treatments. Although water deficit led to gradual declines in Pn, Tr, and Gs, Pn showed a rebound trend under WD + Bic, and the decline in Gs was relatively attenuated. These patterns indicate a divergence in the photosynthetic trajectory of *Bp* under WD + Bic compared with WD alone. Shifts in gas-exchange coordination are commonly reported under water deficit.^36^ Previous studies have shown that environmental stresses rarely act alone; their combined effects can alter cellular ion balance, water relations, and metabolic processes, thereby influencing photosynthetic capacity.^37^

In *Ma*, the temporal response patterns of Pn and Tr differed among treatments, but no recovery was observed under any stress condition. Moreover, gs in *Ma* showed significant main effects of both treatment and time, indicating that the temporal patterns of stomatal conductance were broadly similar across treatments, with no clear evidence that inclusion of bicarbonate in the combined treatment produced an additional stomatal-regulatory pattern under the tested conditions. The interspecific differences in *Bp* and *Ma* observed under WD + Bic may be related to previously reported interspecific differences in photosynthetic adjustment under bicarbonate-associated conditions in karst plants.^38-40^

When photosynthetic dynamics are considered together with growth performance, divergence in photosynthetic trajectories did not necessarily translate into synchronous short-term growth outcomes. *Ma* maintained some growth under water deficit, but growth was more strongly constrained under the combined treatment. In contrast, although some photosynthetic parameters were maintained in *Bp* under WD + Bic conditions, its growth rate still declined. This pattern suggests that under resource-limited conditions, different physiological processes may be differentially prioritized, rather than responding uniformly to stress. Similar decoupling between short-term photosynthetic performance and growth has been documented under water deficit, reflecting shifts in carbon allocation, maintenance costs, and sink limitation.^41^ Therefore, the differences between WD and WD + Bic in early photosynthetic trajectories are better interpreted as whole-plant response patterns under combined stress conditions.

### Hydration adjustment

Previous studies suggest that bicarbonate- or alkali-related conditions can influence plant water relations through shifts in ion balance and osmotic adjustment.^42^ In this experiment, the hydration adjustment patterns in both woody species were different under WD and WD + Bic, and the direction and magnitude of these changes were clearly species-specific. Water deficit significantly reduced leaf water potential in *Bp*, whereas WD + Bic was less reduced than under WD, suggesting a divergence in early hydration trajectory between WD and WD + Bic. In contrast, leaf water potential in *Ma* declined significantly under both WD and WD + Bic, with no apparent recovery under WD + Bic relative to WD. From the time-course perspective of WUE, no treatment effect was detected in *Bp*, whereas *Ma* exhibited a significant treatment × time interaction: the increase in WUE occurred concurrently with suppressed Tr and reduced Pn, consistent with a passive water-saving response.^43^

Electrophysiological water-related indices provide indirect, model-based information on hydration-associated states, because the underlying electrical properties vary with changes in cytoplasmic electrolyte concentration and membrane capacitance under water stress.^44^ In *Bp*, water deficit caused clear deviations of these indices from CK, accompanied by significant increases in IR and IZ, indicating altered electrical properties of leaf tissues, possibly related to changes in ionic movement and membrane-associated characteristics under water deficit.^45^ Under WD + Bic, IWHT shifted partially back toward the control, and IR and IZ showed a decreasing trend, although IXc remained elevated and IC decreased further. Together, these changes indicate that under WD + Bic conditions, the cellular hydration pattern of *Bp* did not simply return toward the pre-stress state, but instead exhibited a dynamic hydration pattern distinct from both the CK and WD-alone treatments. By contrast, *Ma* maintained WTR and intrinsic electrophysiological features close to CK under WD, whereas WD + Bic induced concurrent electrophysiological deviations and downward shifts in WTR and IWC, suggesting that hydration dynamics under WD + Bic diverged from the relatively stable pattern observed under WD alone.^23^^,^^46^

Overall, WD + Bic conditions were associated with species-specific divergence in early-stage water response patterns. *Bp* exhibited a partial hydration rebound relative to WD, whereas *Ma* remained relatively stable under WD but exhibited a more restricted hydration pattern under WD + Bic. These findings indicate that non-destructive electrophysiological indices can help capture and differentiate early hydration response modes among woody species.

### Nutrient dynamics and metabolic energetics

In this study, the nutrient dynamics and energy indices of different species showed different response patterns, not a uniform, one-directional nutrient-limitation response. Because nutrient transport depends on membrane-associated exchange processes, shifts in electrophysiological traits may provide indirect information on nutrient-related responses under stress.^34^^,^^47^ At the nutrient-transport level, although NTR declined in both species under WD + Bic, indicating a general constraint on nutrient transport, the two species differed in their responses under stress. Such declines are frequently linked to reduced xylem transport and diminished root uptake under limited water availability.^48^ In *Bp*, UNF increased significantly under WD while NUE and NAC decreased, suggesting accelerated nutrient turnover at the cost of reduced efficiency. This pattern aligns with the widely discussed trade-off between nutrient turnover and nutrient-use efficiency.^49^ Under WD + Bic, UNF and NUE recovered to control levels and NAC partially rebounded, suggesting a divergence in nutritional turnover–efficiency coordination under WD + Bic relative to WD alone. By contrast, *Ma* showed a stepwise decline in NAC (CK > WD > WD + Bic), whereas NUE and UNF remained largely stable, indicating a decoupling between translocation capacity and nutrient-use efficiency under WD + Bic. Previous studies suggest that bicarbonate-related conditions may constrain nutrient availability and uptake, which may help explain why nutrient-transport patterns diverged between WD and WD + Bic in a species-dependent manner.^50^ Therefore, species-specific nutrient regulation under combined stress was mainly reflected in differential coordination between turnover rate and utilization efficiency.

Nutrient transport is highly dependent on metabolic energy supply. Nutrient uptake and transmembrane ion transport are tightly coupled to ATP-dependent pumps and cellular energy metabolism, making energy status a key constraint on transport under stress.^51^ Therefore, we further examined Δ*G_R_*, Δ*G_Z_*, and Δ*G* as electrophysiologically inferred energetic indices to compare energetic response patterns under stress. Overall, WD reduced Δ*G_R_* and Δ*G* in *Bp*, whereas these indices increased under WD + Bic. The increase in these indices coincided with the partial recovery of Pn observed in *Bp*. By contrast, in *Ma*, Δ*G*_*R*_ showed only a modest increase under WD + Bic without a concurrent rise in Δ*G*, and Pn remained constrained, indicating a different pattern in these energy-related indices compared with *Bp*. Species-specific metabolic responses under combined stresses have been widely reported and may be associated with differences in nutrient-transport patterns.^52^ Overall, under short-term water deficit, nutrient-related electrophysiological indicators and energy-related indices indicated species-specific divergence in response patterns under WD + Bic.

### Integrative comparison of early response modes

Organ-level physiological measures are informative yet can be insufficient on their own for interpreting coordinated cellular adjustments under combined stress. Electrophysiological parameters provide indirect and model-based information related to transmembrane electrical behavior and hydration- or nutrient-associated response patterns.^53^^,^^54^ Thus, these indicators capture treatment-induced shifts in the multivariate electrophysiological state of leaves and highlight species differences in cellular-level response patterning under interacting stresses.

In *Bp*, the CK → WD and CK → WD + Bic centroid shift differed markedly, suggesting that the multivariate displacement of electrophysiology traits under water deficit conditions was not parallel to that under dual stress. This divergence is consistent with the univariate results, suggesting altered coordination among measured response dimensions at the leaf level, rather than a comprehensive return to the control state. By contrast, in *Ma*, the CK → WD and CK → WD + Bic centroid shift was more closely aligned, implying that the overall trait responses under combined stress largely followed the direction of water-deficit-induced responses, with the WD + Bic centroid deviating further from CK than water deficit alone. This interpretation is likewise supported by the corresponding univariate analyses, suggesting that *Ma* relied on a largely similar suite of physiological adjustments under both WD and WD + Bic, but experienced a quantitative exacerbation under the dual constraint.

**Figure 7.**
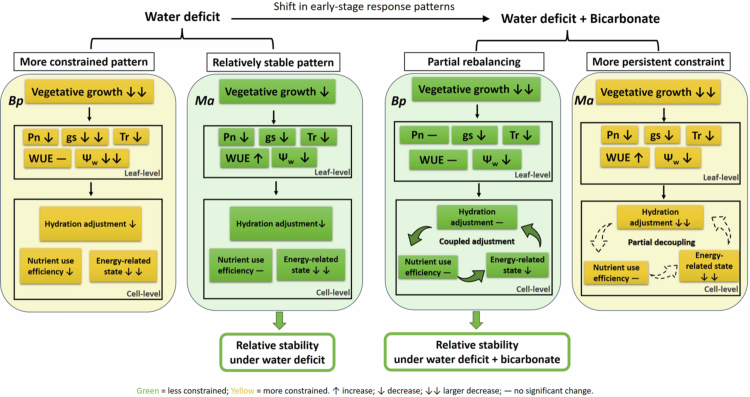
Conceptual synthesis of cross-scale physiological response patterns of *Broussonetia papyrifera* (*Bp*) and *Morus alba* (*Ma*) during the early stage under water deficit and combined water deficit and bicarbonate. Symbols (↑/↓/—) summarize the direction of change relative to the control within each species. Solid vs. dashed curved arrows denote relatively stronger vs. weaker cross-process coordination.

This study systematically compared the integrated, multi-trait responses of *Bp* and *Ma* seedlings under short-term WD and WD + Bic. Under WD + Bic, *Bp* showed partial rebalancing tendencies, manifested by more coordinated directional changes across photosynthesis-, hydration-, nutrient-transport-, and energy-related readouts. In *Ma*, WD caused comparatively modest deviations from CK, but this relative stability under WD alone became less apparent under WD + Bic, indicating more persistent or intensified constraints and weaker cross-process coordination. These patterns indicate that interspecific differences observed under water deficit alone cannot be directly extrapolated to performance when bicarbonate is simultaneously present (Figure 7). Meanwhile, the rapid characterization of water and ion dynamics by electrophysiological approaches suggests their potential as non-destructive candidate tools for early identification of differential response patterns under bicarbonate-co-occurring water-deficit scenarios and for supporting species–site matching in bicarbonate-affected environments.

This study captures early-stage adjustments to PEG-induced water deficit under a bicarbonate co-occurrence scenario relevant to carbonate-rich settings. Accordingly, the present results are best interpreted as evidence of early-stage response patterns under the tested conditions rather than as definitive estimates of treatment effects. Because the design contrasted WD with WD + Bic, our inferences focus on trajectory differences between these treatments rather than on the independent physiological effects of bicarbonate. Although this study employs a biophysical model that approximates mesophyll cells as a concentric spherical capacitor to characterize their electrical properties, it assumes a uniform distribution of ions and idealized membrane characteristics, which may not fully represent the spatial and functional complexity of living plant tissues. Future work incorporating bicarbonate-only controls, expanded replication, and longer-term soil-drying or field trials, together with independent validation using direct physiological measurements (e.g., membrane potential, ion flux, ATP content, transporter abundance, or aquaporin-related traits), will help test the generality of these response modes and strengthen the biological interpretation of these electrophysiological indices.

## Conclusions

This study combined conventional physiological traits with non-destructive electrophysiological phenotyping to characterize early-stage response modes of *Bp* and *Ma* seedlings under a short-term water-deficit assay, with and without bicarbonate. The results indicate that, relative to WD alone, WD + Bic did not uniformly intensify early stress responses but was associated with species-specific shifts in response patterns. Under the combined condition, *Bp* showed a tendency toward rebalancing, with several indicators shifting back toward or remaining close to the control level. In contrast, *Ma* was overall less affected than *Bp* under water deficit alone, but showed more constrained hydration- and transport-associated response patterns under WD + Bic. Electrophysiological indices helped distinguish these differences at the cellular-response level, providing a non-destructive layer for comparing early response modes under compound constraints. Overall, our findings support electrophysiological phenotyping as a candidate tool for early screening and species–habitat matching in carbonate-dominated karst environments, although further validation under longer-term soil-drying regimes and field conditions in karst environments is still required.

## Supplementary materials

Table S1. The 1/2-strength modified composition of Hoagland nutrient solution. Concentrations are final concentrations in 1L solution; Figure S1. Schematic diagram of the parallel-plate. (1) holder; (2) foam spacer; (3) plate electrode; (4) electrical conductor; (5) iron block; (6) plastic rod; (7) bench hold; Table S2. Fitted parameters of Equations (4)–(8) describing the relationships between clamping force and leaf electrical parameters in *Broussonetia papyrifera* and *Morus alba* under three treatments: control (CK), water deficit (WD), and combined water deficit and bicarbonate (WD + Bic). Values are presented as mean (SE), n = 3. All fittings showed *R*^2^ values > 0.98.

## Supplementary Material

Supplementary material Revised_Supplementary_Materials- clean.docx

## Data Availability

All data supporting the findings of this study are included in the article and its supplementary materials.

## Abbreviations

*Bp*: *Broussonetia papyrifera*
*Ma*: *Morus alba*
CK: control
WD: water deficit
WD + Bic: water deficit and bicarbonate
PEG6000: polyethylene glycol 6000
Pn: net photosynthetic rate
gs: stomatal conductance
Tr: transpiration rate
WUE: water use efficiency
Ψ_*w*_: water potential
*R*: resistance
*Z*: impedance
*C*: capacitance
Xc: capacitive reactance
XL: inductive reactance
IR: inherent conduction resistance
IZ: intrinsic impedance
IXc: intrinsic capacitive reactance
IXL: intrinsic inductive reactance
IC: intrinsic capacitance
IWC: intrinsic water conductivity
IWHC: intracellular water-holding capacity
IWHT: intracellular water-holding time
WTR: dynamic water transfer rate
NPT: passive nutrient transport capacity
NAT: active nutrient transport capacity
NUE: nutrient use efficiency
NTR: nutrient transfer rate
UAF: active transport flow of nutrient
NAC: nutrient active translocation capacity
UNF: unit for nutrient-relative transport flow
Δ*G_R_*: metabolic energy of plant leaf cells based on *R*
Δ*G_Z_*: metabolic energy of plant leaf cells based on *Z*
Δ*G*: cellular metabolic energy
