# Supplementary material for: Leaf electrophysiological readouts reveal bicarbonate-associated shifts in early water-deficit response modes in Broussonetia papyrifera and Morus alba
Source: Plant Signal Behav. 2026 Apr 9;21(1):2657093. doi: 10.1080/15592324.2026.2657093 (PMC13078245; doi:10.1080/15592324.2026.2657093)
Supplement: Supplementary material — Revised_Supplementary_Materials- clean.docx [file KPSB_A_2657093_SM3287.docx]

**Supplementary Materials**

**Table S1.** The 1/2-strength modified composition of Hoagland nutrient solution. Concentrations are final concentrations in 1 L solution.

| Component | Concentration (mM) |
| --- | --- |
| KNO_3_ | 3 |
| Ca(NO_3_)_2_·4H_2_O | 2 |
| NH_4_H_2_PO_4_ | 0.125 |
| NH_4_Cl | 0.375 |
| MgSO_4_·7H_2_O | 1 |
| Component | Concentration (μM) |
| H_3_BO_3_ | 25 |
| MnSO_4_·4H_2_O | 2 |
| ZnSO_4_·7H_2_O | 2 |
| CuSO_4_·5H_2_O | 0.1 |
| (NH_4_)_6_Mo_7_O_24_·4H_2_O | 0.1 |
| Fe-EDTA | 50 |


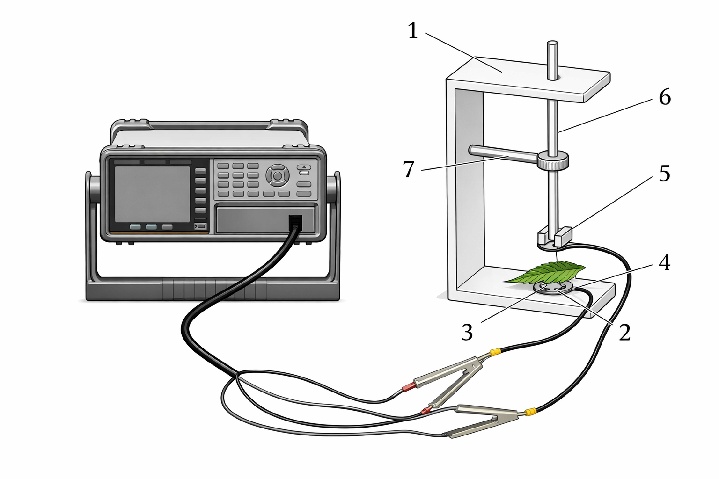


**Figure S1**. Schematic diagram of the parallel-plate. (1) holder; (2) foam spacer; (3) plate electrode; (4) electrical conductor; (5) iron block; (6) plastic rod; (7) bench hold.

**Table S2.** Fitted parameters of Equations (4)–(8) describing the relationships between clamping force and leaf electrical parameters in *Broussonetia papyrifera* and *Morus alba* under three treatments: control (CK), water deficit (WD), and combined water deficit and bicarbonate (WD+Bic). Values are presented as mean (SE), n = 3. All fittings had R^2^ > 0.98.

| Species | Treatment | $R=y_{0}+k_{1}e^{-b_{1}F}$ | | | $Z=p_{0}+k_{2}e^{-b_{2}F}$ | | | $Xc=q_{0}+k_{3}e^{-b_{3}F}$ | | | $XL=a_{0}+k_{4}e^{-b_{4}F}$ | | | $C=x_{0}+hF$ | |
| --- | --- | --- | --- | --- | --- | --- | --- | --- | --- | --- | --- | --- | --- | --- | --- |
|  |  | y_0_ | k_1_ | b_1_ | p_0_ | k_2_ | b_2_ | q_0_ | k_3_ | b_3_ | a_0_ | k_4_ | b_4_ | x_0_ | h |
| *Bp* | CK | 0.07  (0.02) | 0.57  (0.07) | 0.65  (0.04) | 0.04  (0.01) | 0.42  (0.05) | 0.70  (0.06) | 0.06  (0.01) | 0.61  (0.07) | 0.79  (0.10) | 0.14  (0.02) | 1.13  (0.23) | 0.85  (0.20) | -4.15  (5.66) | 157.56  (13.87) |
|  | WD | 0.21  (0.03) | 1.61  (0.13) | 0.55  (0.03) | 0.11  (0.01) | 0.86  (0.15) | 0.63  (0.03) | 0.13  (0.01) | 0.93  (0.13) | 0.61  (0.01) | 0.23  (0.08) | 1.96  (0.16) | 0.50  (0.08) | 22.26  (12.78) | 58.52  (5.09) |
|  | WD+Bic | 0.01  (0.00) | 0.92  (0.03) | 0.28  (0.00) | 0.08  (0.01) | 0.77  (0.10) | 0.56  (0.08) | 0.11  (0.00) | 1.08  (0.12) | 0.65  (0.07) | 0.07  (0.06) | 1.41  (0.05) | 0.34  (0.05) | 0.68  (11.21) | 69.11  (6.58) |
| *Ma* | CK | 0.25  (0.01) | 1.59  (0.08) | 0.73  (0.01) | 0.10  (0.00) | 0.50  (0.03) | 0.67  (0.02) | 0.11  (0.00) | 0.52  (0.04) | 0.66  (0.02) | 0.32  (0.01) | 1.85  (0.11) | 0.71  (0.01) | 78.19  (5.75) | 69.40  (0.81) |
|  | WD | 0.46  (0.07) | 2.17  (0.39) | 0.89  (0.10) | 0.14  (0.03) | 0.69  (0.06) | 0.50  (0.05) | 0.09  (0.03) | 0.76  (0.06) | 0.41  (0.06) | 0.43  (0.14) | 2.66  (0.43) | 0.80  (0.18) | 37.86  (6.06) | 44.12  (2.24) |
|  | WD+Bic | 0.60  (0.10) | 5.06  (0.69) | 0.58  (0.11) | 0.19  (0.02) | 0.99  (0.21) | 0.53  (0.07) | 0.20  (0.01) | 1.04  (0.20) | 0.54  (0.06) | 0.75  (0.08) | 7.66  (2.23) | 0.65  (0.14) | 33.41  (9.00) | 32.55  (2.65) |
